# Updating The General Practitioner on The Association Between Teeth Loss and Temporomandibular Disorders: A Systematic Review

**DOI:** 10.1055/s-0042-1757209

**Published:** 2022-12-27

**Authors:** Marília da Cunha Feio Leal, Micaele Maria Lopes Castro, Márcia Consentino Kronka Sosthenes

**Affiliations:** 1 Laboratório de Investigações em Neurodegeneração e Infecção, Instituto de Ciências Biológicas, Hospital Universitário João de Barros Barreto, Universidade Federal do Pará, Belém, Brazil

**Keywords:** tooth loss, dental occlusion, temporomandibular joint, systematic review

## Abstract

The belief about a possible association between the absence of one or more teeth and the presence of temporomandibular disorders (TMD), although old, is still present among the dental class. Although evidence points to a lack of association between loss of posterior support and the presence of TMD, we do not have critical studies on the extent, quantity, or location of these losses. In this sense, this systematic review aims to investigate the association between tooth loss and the presence of TMD signs or diagnostic subgroups. Search strategies using a combination of keywords tooth loss and TMDs were performed in six databases (PubMed, Embase, Web of Science, Livivo, Lilacs, and Scopus) and gray literature from August to September 2020. Observational studies that investigated the association between tooth loss in TMD were considered. The risk of bias was assessed using the Joanna Briggs Institute (JBI) Critical Assessment Checklist for cross-sectional analytical studies, case–control, and cohort studies. Finally, the level of certainty measured by the Grading of Recommendations Assessment, Development, and Evaluation (GRADE) was assessed. Six articles were included in the review according to the eligibility criteria. Of these, five had a high risk of bias and one had a moderate risk. Only one study showed an association between the loss of posterior teeth and the presence of joint sounds and joint pain, the others found no significant association with sign or TMD subgroups diagnostic.

There is no scientific evidence to support the association between one or more tooth loss and the presence of TMD signs and symptoms or diagnostic subgroups.

## Introduction

 Despite the reduction of prevalence and overall incidence of edentulism, tooth loss still represents a public health problem in several countries especially in the older population. 1 2 3 4 Further, it is important to note that demographic and socioeconomic factors referring to low income seem to contribute to the condition. 4 5 6 7

 In addition to affecting the quality of life, 8 impairing occlusal balance, and masticatory capacity, 9 tooth loss has been considered a predictor or risk factor for cognitive decline and dementia. 10 There are also records of a possible association of this condition with the presence of signs and symptoms of temporomandibular dysfunction. 11 12 13 14 15 16 17 18

 Temporomandibular disorders (TMD) is a common complaint among patients attending dental clinic 19 and represents a set of musculoskeletal conditions affecting bone, fibrous, cartilaginous, and\or muscular structures involved in mandibular movements. It is characterized by the presence of one or more symptoms such as pain at pre-auricular region, face or temple, limiting movement, raising up joint noise, among others. 20 However, its pathophysiology is still poorly understood and throughout decades it has been investigated. 21

 Until the 1950s, TMD etiological understanding was based purely on gnathological and mechanistic theories linked to dental occlusion; from this period on, concepts were modified and more complex, aggregating multifactorial etiology and biopsychosocial characteristics are gaining ground and establishing itself in the literature. 22 23

 Although studies on the possible relationships between tooth loss and the presence of TMD signs and symptoms are old, 24 there is so far no systematic critical analysis with direction and focus in these studies. Despite of the evidence pointing to a lack of association between loss of posterior support and TMD, there is no mention of the extent of the support, or the number of missing teeth, or even the distribution of the remnants in the arch. 25

 Although the fact that the current evidence encourages dentists to abandon the gnathological paradigms related to the role of occlusion in etiology and treatment of TMD, 25 26 the belief that tooth loss may be associated with the presence of TMD has still been frequent among them. 19 27 For this reason, the purpose of this systematic review is to critically evaluate studies on the subject and answer the question: Is there any association between the loss of one or more teeth and the presence of temporomandibular dysfunction? 

## Methods

### Protocol and Registration

 This systematic review was registered at PROSPERO under the code CRD42020203754 and it was performed according to Preferred Reporting Items for Systematic Reviews and Meta-Analyses (PRISMA) 28 29 ( Supplementary Material Table S1 , available in the online version). 

### Eligibility Criteria

In the present review, we aimed to answer the following question: “Is there an association between tooth loss and temporomandibular disorders (TMDs)?” The eligibility criteria was defined according to PECO strategy in which the acronym “P” represents the Patient, "E” stands for Exposition, "C” for Comparison and O for outcome characteristics for the eligible question. Only observational studies employing adult human population (P), which evaluate exposed (E) and non-exposed patients to tooth loss (C) and assess the association between tooth loss and outcomes related to TMDs (O) were included in this review. These included studies were published in indexed journals without restriction of year of publication or language to obtain a very broad research covering as many studies as possible about the subject.

 Exclusion criteria were defined as the use of dental prosthesis, absence of patient clinical exam as well as narrative reviews, case reports, descriptive studies, technical articles, animal, child and *in vitro* studies. 

### Information Sources

 The searches were performed on following electronic databases: PubMed, Scopus, Web of Science, LILACS and Cochrane Library. Google Scholar and The Open Grey were used as gray literature sources. No restriction of year or language was applied. The search strategy was composed by MESH and entry terms adapted according to each database, using Boolean operators (OR, AND) to combine the searches ( Table 1 ). 

**Table 1:** Search strategies in different databases

Database	Search	Records
EMBASE	#1 “craniomandibular disorders”/exp OR “craniomandibular disorders” OR “temporomandibular joint disorders”/exp OR “temporomandibular joint disorders” OR “temporomandibular joint dysfunction syndrome”/exp OR “temporomandibular joint dysfunction syndrome” OR “disorders, temporomandibular joint” OR “joint disorder, temporomandibular” OR “joint disorders, temporomandibular” OR “myofascial pain dysfunction syndrome”/exp OR “myofascial pain dysfunction syndrome” OR “temporomandibular joint”/exp OR “temporomandibular joint” OR “tmj syndrome” OR “syndrome, tmj” OR “temporomandibular joint syndrome”/exp OR “temporomandibular joint syndrome” OR “joint syndrome, temporomandibular” OR “syndrome, temporomandibular joint” OR “craniomandibular disorder” OR “disorder, craniomandibular” OR “disorders, craniomandibular[ OR “craniomandibular diseases” OR “disease, craniomandibular” OR “diseases, craniomandibular” #2 “tooth loss”/exp OR “tooth loss”/de OR “mouth, edentulous”/exp OR “mouth, edentulous”/de OR “jaw, edentulous”/exp OR “jaw, edentulous”/de OR “loss, tooth” OR “edentulous mouth” OR “edentulous mouths” OR “mouth, toothless” OR “toothless mouth” OR “edentulous jaw”/exp OR “edentulous jaw”/de OR “edentulous jaws” OR “jaws, edentulous” OR “edentulism”/exp OR “edentulism”/de OR “dental occlusion”/exp OR “dental occlusion”/de OR “edentulousness”/exp OR “edentulousness”/de	671
LILACS	tw:((tw:(“Trastornos de la Articulación Temporomandibular” OR “Transtornos da Articulação Temporomandibular” OR “Síndrome de la Disfunción de Articulación Temporomandibular” OR “Síndrome da Disfunção da Articulação Temporomandibular” OR “Articulación Temporomandibular” OR “Articulação Temporomandibular”)) AND (tw:(“Pérdida de Diente” OR “Perda de Dente” OR “Boca Edéntula” OR “Boca Edêntula” OR “Arcada Edéntula” OR “Arcada Edêntula” “Arcada Desdentada” OR “Maxila Edentada” OR “Maxilar Desdentado” OR “Maxilar Edentado” OR “Maxilar Edêntulo” OR “Arcada Parcialmente Edêntula” OR “Arcada Parcialmente Edéntula” OR “Oclusión Dental” OR “Oclusão Dentária”))) AND (db:(“LILACS”))	236
LIVIVO	“Tooth Loss” OR “Edentulism” OR “Edentulousness” AND ”Craniomandibular Disorders” OR “Temporomandibular Joint Disorders” OR “Temporomandibular Joint Dysfunction Syndrome” OR “Myofascial Pain Dysfunction Syndrome” OR “Temporomandibular Joint Syndrome” OR “Joint Syndrome, Temporomandibular” OR “Syndrome, Temporomandibular Joint” OR “Craniomandibular Disorder” OR “Craniomandibular Diseases”	3214
PubMed	((((((((((((((((((Tooth Loss[MeSH Terms]) OR (tooth loss[Title/Abstract])) OR (Mouth, Edentulous[MeSH Terms])) OR (Mouth, Edentulous[Title/Abstract])) OR (Jaw, Edentulous[MeSH Terms]))) OR (Loss, Tooth[Title/Abstract])) OR (Edentulous Mouth[Title/Abstract])) OR (Edentulous Mouths[Title/Abstract])) OR (Mouth, Toothless[Title/Abstract])) OR (Toothless Mouth[Title/Abstract])) OR (Edentulous Jaw[Title/Abstract])) OR (Edentulous Jaws[Title/Abstract])) OR (Jaws, Edentulous[Title/Abstract])) OR (Edentulism[Title/Abstract])) OR (dental occlusion[MeSH Terms])) OR (dental occlusion[Title/Abstract])) AND ((((((((((((((((((((((((((Craniomandibular Disorders[MeSH Terms]) OR (Craniomandibular Disorders[Title/Abstract])) OR (Temporomandibular Joint Disorders[MeSH Terms])) OR (Temporomandibular Joint Disorders[Title/Abstract])) OR (Temporomandibular Joint Dysfunction Syndrome[MeSH Terms])) OR (Temporomandibular Joint Dysfunction Syndrome[Title/Abstract])) OR (Disorders, Temporomandibular Joint[Title/Abstract])) OR (Joint Disorder, Temporomandibular[Title/Abstract])) OR (Joint Disorders, Temporomandibular[Title/Abstract])) OR (Myofascial Pain Dysfunction Syndrome,[Title/Abstract])) OR (Temporomandibular Joint[Title/Abstract])) OR (TMJ Syndrome[Title/Abstract])) OR (Syndrome, TMJ[Title/Abstract])) OR (Temporomandibular Joint Syndrome[Title/Abstract])) OR (Joint Syndrome, Temporomandibular[Title/Abstract])) OR (Syndrome, Temporomandibular Joint[Title/Abstract])) OR (Craniomandibular Disorder[Title/Abstract])) OR (Disorder, Craniomandibular[Title/Abstract])) OR (Disorders, Craniomandibular[Title/Abstract])) OR (Craniomandibular Diseases[Title/Abstract])) OR (Disease, Craniomandibular[Title/Abstract])) OR (Diseases, Craniomandibular[Title/Abstract]))	2673
Scopus	TITLE-ABS-KEY (“Tooth Loss” OR “Mouth, Edentulous” OR “Jaw, Edentulous” OR “Loss, Tooth” OR “Edentulous Mouth” OR “Edentulous Mouths” OR “Mouth, Toothless” OR “Toothless Mouth” OR “Edentulous Jaw” OR “Edentulous Jaws” OR “Jaws, Edentulous” OR “Edentulism” OR “Dental occlusion” OR “Edentulousness”) AND TITLE-ABS-KEY (“Craniomandibular Disorders” OR “Temporomandibular Joint Disorders” OR “Temporomandibular Joint Dysfunction Syndrome” OR “Disorders, Temporomandibular Joint” OR “Joint Disorder, Temporomandibular” OR “Joint Disorders, Temporomandibular” OR “Myofascial Pain Dysfunction Syndrome” OR “Temporomandibular Joint” OR “TMJ Syndrome” OR “Syndrome, TMJ” OR “Temporomandibular Joint Syndrome” OR “Joint Syndrome, Temporomandibular” OR “Syndrome, Temporomandibular Joint” OR “Craniomandibular Disorder” OR “Disorder, Craniomandibular” OR “Disorders, Craniomandibular[“ OR “Craniomandibular Diseases” OR “Disease, Craniomandibular” OR “Diseases, Craniomandibular”) AND (LIMIT-TO (DOCTYPE, “ar”)) AND (EXCLUDE (SUBJAREA, “COMP”) OR EXCLUDE (SUBJAREA, “ENGI”) OR EXCLUDE (SUBJAREA, “HEAL”) OR EXCLUDE (SUBJAREA, “VETE”) OR EXCLUDE (SUBJAREA, “ARTS”))	2724
Web of Science	TÓPICO: (“Tooth Loss” OR “Mouth, Edentulous” OR “Jaw, Edentulous” OR “Loss, Tooth” OR “Edentulous Mouth” OR “Edentulous Mouths” OR “Mouth, Toothless” OR “Toothless Mouth” OR “Edentulous Jaw” OR “Edentulous Jaws” OR “Jaws, Edentulous” OR “Edentulism” OR “Dental occlusion” OR “Edentulousness”) *AND* TÓPICO: (”Craniomandibular Disorders” OR “Temporomandibular Joint Disorders” OR “Temporomandibular Joint Dysfunction Syndrome” OR “Disorders, Temporomandibular Joint” OR “Joint Disorder, Temporomandibular” OR “Joint Disorders, Temporomandibular” OR “Myofascial Pain Dysfunction Syndrome” OR “Temporomandibular Joint” OR “TMJ Syndrome” OR “Syndrome, TMJ” OR “Temporomandibular Joint Syndrome” OR “Joint Syndrome, Temporomandibular” OR “Syndrome, Temporomandibular Joint” OR “Craniomandibular Disorder” OR “Disorder, Craniomandibular” OR “Disorders, Craniomandibular[“ OR “Craniomandibular Diseases” OR “Disease, Craniomandibular” OR “Diseases, Craniomandibular”)	286
Google Scholar	(“Temporomandibular Disorders” OR Temporomandibular Joint Disorders) AND (“tooth loss” OR “edentulous mouth”)	100
OpenGrey	“Tooth Loss” OR “Mouth, Edentulous” OR “Jaw, Edentulous” OR “Loss, Tooth” OR “Edentulous Mouth” OR “Edentulous Mouths” OR “Mouth, Toothless” OR “Toothless Mouth” OR “Edentulous Jaw” OR “Edentulous Jaws” OR “Jaws, Edentulous” OR “Edentulism” OR “Dental occlusion” OR “Edentulousness” AND ”Craniomandibular Disorders” OR “Temporomandibular Joint Disorders” OR “Temporomandibular Joint Dysfunction Syndrome” OR “Disorders, Temporomandibular Joint” OR “Joint Disorder, Temporomandibular” OR “Joint Disorders, Temporomandibular” OR “Myofascial Pain Dysfunction Syndrome” OR “Temporomandibular Joint” OR “TMJ Syndrome” OR “Syndrome, TMJ” OR “Temporomandibular Joint Syndrome” OR “Joint Syndrome, Temporomandibular” OR “Syndrome, Temporomandibular Joint” OR “Craniomandibular Disorder” OR “Disorder, Craniomandibular” OR “Disorders, Craniomandibular“ OR “Craniomandibular Diseases” OR “Disease, Craniomandibular” OR “Diseases, Craniomandibular”	89

 The searches were performed on August 18, 2021 ( Supplementary Material Table S2 , available in the online version). Additionally, an alert was created in each database to retrieve new studies according to eligibility criteria. 

### Selection and Data Collection Process

The completed search results were downloaded into Endnote X8 for citation management and deduplicated (EndNote, X9 version, Thomson Reuters, Philadelphia, United States). Screening was done in Rayyan, a web-based literature screening program.

All evaluations, including searches, study selections, data extraction, and bias evaluation risk were performed independently by two reviewers (M.C.F.L. and M.M.L.C. and verified by a third appraiser in case of disagreements—M.C.K.S.).

### Data Items

After selection process, following data were extracted from the included studies: methodological design, country, publication year, sample general characteristics, patient ages, tooth loss classification method, TMD classification method, statistical analysis, main results and conclusions.

### Risk of Bias in Individual Studies

 Selected articles were critically assessed by the same reviewers using JBI Critical Appraisal Checklist from Joanna Briggs Institute. 30 This qualifying method was used for both cross-sectional and cohort studies ( Supplementary Material Table S3 , available in the online version) and the articles that reflected the purpose of the present investigation according to opinion of one or both reviewers were analyzed in full, and a common consensus was reached (M.C.F.L. and M.M.L.C.). 

Then, two reviewers (M.C.F.L. and M.M.L.C.) separately performed the risk of bias evaluation and judge included articles as “high risk” when the study reaches up to 49% score “yes,” “moderate risk” when the study reached 50 to 69% score “yes,” and “low risk” when the study reached more than 70% score “yes.” A conference between the two reviewers was made, and any discordance was discussed and decided with a third review (M.C.K.S.)

### Level of Evidence

 The level of evidence was interpreted according to the Grading of recommendations (R), assessment (A), development (D), and evaluation (E) (GRADE) approach, with a narrative evaluation. 31 This tool aimed to summarize the evidence tracked, based on the four steps and considering the risk of bias, inconsistency, indirectness, imprecision focusing on the certainty of evidence among included studies in the systematic review. 

## Results

**Fig. 1:**
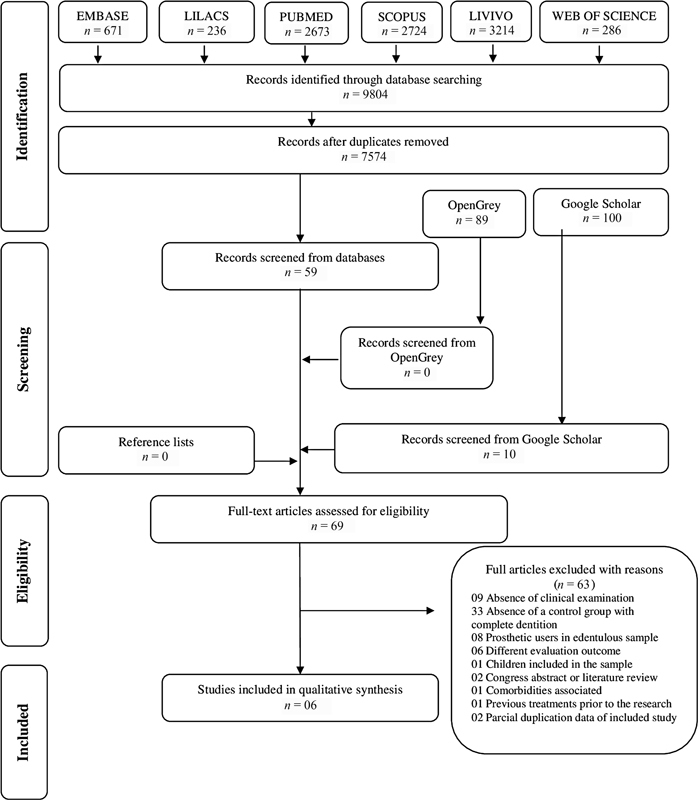
Flow diagram of literature search and selection criteria including the following phases: identification, screening, and eligibility included.

 After a broad search on databases, a total of 9,804 articles were found. Removing duplicates, 7,754 articles remained for title and abstract reading, and, 69 were selected according to the eligibility criteria for full reading. The excluded studies in full text reading phase are available at the Supplementary Material Table S4 (available in the online version) and no additional articles were cited at reference list. At Fig. 1 , a flowchart describes selection process of publications in the respective databases. 

### Study Characteristics

 Of the 69 selected articles, only six were included in the present review, and reasons related to elections were described at Fig. 1 . Among the six selected articles, one was a cohort, 32 two case–controls, 33 34 and three as transversal type. 35 36 37 Two researches were carried out in Brazil, 35 37 two at the Netherlands, 32 34 one in Iran, 33 and one in Mexico 36 considering their nationality origin. One of the main reasons for exclusions was absence of control group with complete dentition being compared to tooth loss group. In most articles, control group was formed by individuals who still had some tooth loss. The use of removable prosthesis by participants and lack of clinical examination for TMD diagnosis were also reasons for exclusion. If the author clearly cited the use of removable prosthesis by the research participants, this study was excluded due to the potential risk of bias in assessing the effect of tooth loss and DTM. The summary of these results is described in Table 2 . 

**Table 2:** Summary of characteristics of the included studies ( *n*  = 06)

Study design	Author, Years, Country	Sample *N* (gender)	Sample characteristics	Groups ( *n* )	Age (mean ± SD or range in years)	Tooth loss assessment classification	TMD Diagnostic method/ objectives	Statistical analysis	Results (mean ± SD, or other pertinent findings)	Main conclusion
Case–control	Gil, *1995 Brazil*	102 78 women 24 men	Patients with TMD symptoms convenience sample	a. Class II Kennedy patients with removable partial prosthesis ( *n* = 34) b. Class II Kennedy patients without prosthesis ( *n* = 34) c. Complete dentition ( *n* = 34)	17–61 y	Kennedy Classification	Anamnesis + Clinical examination Evaluated articular sounds (clicking and creptation)	● Kruskal-Wallis complemented with Dunn's post test ● Mann-Whitney 0.05 significance level 95% confidence interval	a. clicking( *n* = 41.1%) creptation ( *n* = 36.8%) total ( *n* = 38.9%) b. Clicking ( *n* = 35.3%) Creptation ( *n* = 23.5%) Total ( *n* = 29.4%) *p* = 0.05	There was a prevalence of sounds for unilateral edentulous patients without prosthesis in relation to those with complete dentition, but with no statistical difference
Case–control	Sarita *et al, 2003* *Netherlands*	725	Subjects with shortened dental arch (SDA)	a. Slightly SDA (at least first molars bilaterally present) ( *n* = 128) b. SDA I and asymmetric SDA I (bilateral premolar and unilateral molar support) ( *n* = 195) c. SDA II and extreme SDA I (bilateral [reduced] premolar support) ( *n* = 194) d. Extreme SDA II (no posterior support) ( *n* = 105) e. Asymmetric SDA II and III (unilateral posterior support) ( *n* = 103) f. Complete dental arches (control) ( *n* = 125) Each group was divided in: ● Younger age group (≥20 <40) and ● Older age group (≥40)	≥20 y	SDAs were identified based on arch length and symmetry	Anamnesis + Clinical examination Evaluated articular sounds (clicking or crepitation) and limited mouth opening <40 mm	● Chi-square test with Bonferroni correction ● Logistic regression analysis 0.05 significance level	Clicking or crepitation in SDA groups without statistical difference: a. ( *n* = 20/16%) b. ( *n* = 24/12%) c. ( *n* = 22/11%) d. ( *n* = 24/23%) e. ( *n* = 19/18%) f. ( *n* = 15/12%) Clicking or crepitation according age groups: ● Older age group (≥ 20 <40): (19%) ● Younger age group (≥40): (9%) *p* < 0.001	Only the complete absence of posterior occlusal support unilaterally or bilaterally appears to increase the risk for developing signs and symptoms associated with TMD, but it has not been statistically confirmed
Cross-sectional	Casanova-Rosado et al, 2006 *Mexico*	506 274 women 232 men	University students		14–25 y Mean: 17.2 ± 2.7	At least one tooth loss	RCD Evaluate presence or absence of TMD	● Bivariate logistical regressions ● Multivariate logistic regression when *p* <0.02 in bivariate analysis 95% confidence interval	At least one tooth loss: *p* = 0.3 High levels of stress + at least one tooth loss OD = 2.4 CI = 1.01–5.9 *p* = 0.04	The effect of stress on TMD depends on the tooth loss, controlling for sex, bruxism, unilateral chewing, and anxiety.
Cohort	Witter et al, 2007 *Netherlands*	146 82 women 64 men	Subjects with shortened dental arch (SDA) convenience sample	Baseline a. SDA group shortened dental arch ( *n* = 74) b. CDA group complete dental arch ( *n* = 72) After 9 y a. SDA ( *n* = 42) b. CDA ( *n* = 41)	Baseline a. SDA: 40.5 ± 11.8 y b. CDA: 36.2 ± 9.8 y After 9 ya. SDA: 41.8 ± 10 y b. CDA: 38.5 ± 9.8 y	Shortened dental arches with intact anterior regions and a variation of occlusal support (3–5 occlusal units in the posterior area)	Questionnaire + clinical examination Evaluated articular sounds (clicking or crepitation) and limited mouth opening <40 mm and reported pain.	Chi-square or *t* -test (baseline) Pearson correlation (follow-up)	Baseline data on symptoms and signs were not significantly different between SDA and CDA groups Cliking\crepitus SDA: 0.55 (0.55) CDA: 0.49 (0.49) Mouth limited opening SDA: 0.36 (0.36) CDA: 0.16 (0.31) Pain reported SDA: 0.24 (0.04) CDA: 0.16 (0.05) After 9 y, 69-79% of the subjects in the SDA group and 70–75% in the CDA group did not report any symptom at separate observations.	Subjects with SDA had similar prevalence, severity, and fluctuation of signs and symptoms related to TMD compared to subjects with complete dental arch.
Case–control	Fallahi et al, 2016 Iran	200 120 women 80 men	Population study	a. Partially edentulous subjects Kennedy Cl I or Cl II ( *n* = 100) b. Complete dentition subjects	18–70 y Mean age a. 45.7 b. 32.7	Kennedy and Eichner classification	Fonseca's questionnaire + Clinical examination Evaluated presence or absence of clicking, joint pain, deviation form path, joint locking	Chi-square test *t* -test	Frequency of TMD (Kennedy) a. 58% b. 43% ( *p* <0.03) Clicking a. 38% b. 19% ( *p* <0.001) Joint pain a. 24% b. 8% ( *p* <0.01) Frequency of TMD (Eichner) increased with decrease in occlusal support area ( *p* <0.02)	Partial edentulism can be an important factor in the induction of TMJ disorders
Cross-sectional	Costa Dutra *et al, 2019, Brazil*	30 26 women, 4 men	Patients with TMD symptoms convenience sample	a. Complete dentition b. Partially edentulous with posterior contention c. Partially edentulous without posterior contention d. Complete edentulous with denture	>18 y	Partially or total edentulousness	RCD Evaluated presence or absence of TMD	● Chi-square test ● Prevalence ratio 0.05 significance level 95% confidence interval	a. Patients with TMD and complete dentition ( *n* = 10). b. Patients with TMD and partially edentulous without posterior contention ( *n* = 4). *p* = 1.0 RP = 1.27 IC = 0.434–3.737	There was no statistically significant association between variables gender, age and dental condition with TMD. It seems that isolated factors don't have influence on the etiological process of TMD

Abbreviations: CDA, completed dental arch; CI, confidence interval; OD, odds ratio; RCD, research criteria diagnostic; SDA, shortened dental arch; TMD, temporomandibular disorders.

### TMD Diagnosis and Classification of Tooth Loss

 From six included studies, four of them used questionnaires and clinical examination not scientifically validated for the diagnosis of TMD 32 34 35 and so only two studies used the research criteria diagnostic (RCD). 36 37

 Regarding classification of tooth loss, most studies evaluated influence of posterior tooth loss on TMD 32 33 34 35 37 and only one research evaluated absence of at least one tooth in dental arch, but did not mention location and/or quantity of tooth loss. 36 The authors used their own criteria to classify different variations in absence of dental elements; only two studies used the Kennedy Classification and one of them also included the Eichner Classification. 33 35 The others researchers considered possible variations within a reduced dental arch which means the absence of posterior support 34 or considered posterior tooth loss, regardless of remnant distribution in dental arch. 32 37

### Risk of Bias

 Only one study was evaluated with a moderate risk of bias, 34 and the others were classified as a high risk of bias. The main factors that led to this evaluation were lack of identification and control of confounding factors, absence of reliable and validated methods for TMD diagnosing, and classification of tooth loss and absence of inclusion and exclusion criteria in the selection of sample groups. The results of bias analysis for cohort, case–control, and cross-sectional study are available in the following tables, respectively ( Tables 3 , 4 , and 5 ). 

**Table 3:** JBI critical appraisal checklist for cohort study

Fallahi et al, 2016	Sarita et al, 2003	Case–control
−	+	Were the groups comparable other than presence of disease in cases or absence of disease in controls?
−	+	Were cases and controls matched appropriately?
+	+	Were the same criteria used for identification of cases and controls?
−	−	Was exposure measured in a standard, valid and reliable way?
+	+	Was exposure measured in the same way for cases and controls?
−	−	Were the confounding factors identified?
−	+	Were the strategies to deal with confounding factors stated?
−	−	Were outcomes assessed in a standard, valid, and reliable way for cases and controls?
**NA**	NA	Was the exposure period of interest long enough to be meaningful?
−	+	Was appropriate statistical analysis used?

Source: Reproduced with permission of Witter et al 2007. ^32^

**Table 4:** JBI critical appraisal checklist for cohort study case–control studies (Fallahi et al 2016; Sarita et al 2003)

Witter et al, 2007	Cohort
+	Were the two groups similar and recruited from same population?
+	Were the exposures measured similarly to assign people to both exposed and unexposed groups?
−	Was the exposure measured in a valid and reliable way?
−	Were the confounding factors identified?
−	Were the strategies to deal with confounding factors stated?
−	Were the groups/participants free of outcome at the beginning of the study (or at the moment of exposure)?
−	Were the outcomes measured in a valid and reliable way?
+	Was the follow-up time reported and sufficient to be long enough for outcomes to occur?
−	Was follow-up complete, and if not, were the reasons to loss to follow-up described and explored?
−	Were strategies to address incomplete follow-up utilized?
+	Was appropriate statistical analysis used?

**Table 5:** JBI critical appraisal checklist for cross sectional studies

Casanova-Rosado et al, 2006	Costa Dutra et al, 2019	Gil, 1995	Cross sectional
−	+	−	Were the criteria for inclusion in sample clearly defined?
−	−	−	Were the study subjects and the setting described in detail?
−	+	+	Was the exposure measured in a valid and reliable way?
−	+	+	Were objective, standard criteria used for the measurement of condition?
+	−	−	Were the confounding factors identified?
+	−	−	Were strategies to deal with confounding factors stated?
+	−	−	Were the outcomes measured in a valid and reliable way?
+	−	+	Was appropriate statistical analysis used?

Source: Reproduced with permission of Casanova-Rosado et al 2006 ^36^ ; Costa Dutra et al 2019 ^37^ ; and Gil 1995. ^35^

 In cohort study of Witter et al, it was noted that the confounding factor was not reported in the research. Moreover, no strategies to deal with possible confounding factors were evaluated in this cohort study. 32

 In case–control studies of Fallahi et al and Sarita et al, they used the same criteria for identification and measurement of variables both for the case and control group. 33 34 Besides, in this study design, the identification of confounding factors was not carried out. 

 In cross sectional studies of Casanova-Rosado et al, Costa Dutra et al, and Gil et al, the subjects were not described in detail. However, Costa Dutra et al and Gil measured the exposure in a valid and reliable way, using clinical examination, signs, and symptoms of DTM and RCD. 35 36 37 Only Casanova et al identified confounding factors and strategies to deal with this distortion. 36

### Results from the Studies

 Gil investigated the prevalence of joint sounds (clicking/crepitation) in a group of 102 individuals with loss of posterior teeth and compared to a complete dentition group. After statistical analyses using the Kruskal-Wallis and Mann-Whitney tests, the author concluded that although the prevalence of these joint noises was higher in the tooth loss group, there was no statistical difference between the groups ( *p* =0.058). 35

 Sarita et al evaluated the presence of joint noises (clicking/crepitation) and restricted mouth opening in individuals with posterior tooth loss ( *n*  = 600), comparing them to complete dentition group ( *n*  = 125). The Chi-square test and logistic regression were performed and no statistical significance between groups was detected, but symptoms were more prevalent in group aged over 40 years ( *p* = 0.001). 34

 Casa Nova et al studied the prevalence of TMD in 506 university students. They used the DC/TMD as criteria diagnostic tool. The loss of at least one dental element was assessed with other possible TMD risk factors (bruxism, stress, unilateral chewing, and anxiety behavior). Logistic regression tests showed an interaction between tooth loss and presence of stress as a risk factor for TMD ( *p*  = 0.04). However, when tooth loss was alone assessed, no statistically significant results were found ( *p*  = 0.3) for any subtype of TMD. 36

 Witter et al carried out a cohort study where individuals with reduced arch ( *n* = 74) and with complete dentition ( *n*  = 72) were followed up to 9 years. During this period, researchers investigated presence of joint noise and restricted mouth opening. The analysis of covariance using a mixed model did not reveal any significant difference between groups regarding presence of related symptoms ( *p* >0.05). The results showed that prevalence, severity, and fluctuation of TMD symptoms were similar in both groups. 32

 Fallahi et al evaluated the presence of signs and symptoms of TMD in individuals with partial edentulism ( *n*  = 100) and compared it to those with complete dentition ( *n*  = 100). The subjects were evaluated for the presence of joint sounds, restricted mouth opening, joint locking, mandibular deviations, joint pain, condylar , and masticatory muscle pain. Chi-square analysis showed that partial edentulism may be an important factor for TMDs ( *p* <0.03) and that TMD frequency increases with decreasing posterior occlusal support ( *p* <0.02). 33

 Costa-Dutra et al evaluated the association between partial tooth loss and the presence of TMD in 30 patients examined by RCD/TMD. The statistical test used was the Chi-square test and the results did not reveal any significant association between the tooth loss and TMD subtypes ( *p*  = 1.0). 37

 In summary from all studies included, only one showed a positive association between tooth loss and presence of signs of TMD. 33 This study evaluated individuals without posterior teeth (Kennedy Class I or II classification) and showed a higher frequency of joint noise ( *p* <0.001) and joint pain ( *p* <0.01) in these groups when compared to a control group with complete dentition. 25 The other studies showed no statistical association with any TMD subtype (axis I, II, or II from RCD /TMD) 28 29 or any assessed signs (joint sounds, restricted mouth opening, and pain). 24 26 27 The summary with individual data for each article is described in Table 2 . 

### Assessment of Certainty of Evidence

 The certainty of evidence was assessed in conjunction with the present six included studies and proved to be very low according to the GRADE criteria. This was due to serious bias risk of imprecision and very serious inconsistency found at the related research ( Table 6 ). It was not possible to perform a meta-analysis in this review due to high heterogeneity found in variation of methods used to classify tooth loss, TMD diagnosis, and differences in effect estimates of statistical analyses. 

**Table 6:** Evidence summaries from Grading of Recommendations Assessment, Development and Evaluation (GRADE)

Certainty assessment							Impact	Certainty
N° of studies	Study design	Risk of bias	Inconsistency	Indirectness	Imprecision	Other considerations		
6	Observational studies	Serious a	Extremely serious b	Not serious	Serious c			⊕◯◯◯very low

a The risk of bias for all articles was high, with the exception of one (Sarita et al, 2003) which proved to be moderate.

b High heterogeneity demonstrated in outcomes of the studies with regard to effect characteristics, diagnostic criteria for TMD, classification of tooth loss and different groups.

c Estimates of effect were not found in most studies, a small number of events in half of the studies.

## Discussion

 Observational studies that investigated the relationship between tooth loss and the presence of TMD or signs and symptoms of this disorder were selected by eligibility criteria. Most of these studies were excluded ( *n*  = 53) because they did not present a control group of dentate patients or because they did not include clinical examination in the evaluation of their sample. At the end, only six studies 32 33 34 35 36 37 were included in this review, being three cross-sectional, 35 36 37 two case–control, 33 34 and one cohort. 32 Each article was individually evaluated by the authors in relation to its methodological quality using the JBI Critical Appraisal Tools. Subsequently, they were evaluated together for the risk of bias by the GRADE system, 31 and were classified with a very low certainty of evidence due to a high risk of bias characterized by the lack of randomization of samples, lack of blinding, confounding bias, and selection bias. The inconsistency was extremely serious as most of them presented results without appropriate association measures or omission of confidence intervals, as well as unrepresentative samples of the population, which also compromised precision and indirectness. Of these six studies, 32 33 34 35 36 37 only one of them showed an association between posterior tooth loss and the presence of joint clicks and temporomandibular joints (TMJs) pain, 33 the others did not show any association between the types of tooth loss evaluated and the diagnosis of TMD or presence of signs and symptoms. 

 TMD is an umbrella term for pain and dysfunction involving the masticatory muscles and TMJs. 38 This complex disorder results from interaction of multiple causes with genetic and environmental domains. ^39^ The loss of teeth has long been investigated as a possible association with signs and symptoms of TMD 40 and this belief has persisted over years, 41 although its cause–effect relationship has never been proven. An association between these conditions emerged in times when reliable and valid protocol for assessing patients with TMD did not exist, i.e., it got introduced from the RCD/TMD only in 1992. 42

 Of the six studies included in this review, only two of them used RCD as a diagnostic criteria. 36 37 In these observational studies, no association was found between the presence of TMD and the loss of one or more teeth, 36 or between the partial loss of teeth regardless of occlusal support. 37 A positive relationship between these conditions was found when tooth loss was evaluated alone (OR: 1.3), but when evaluated together with other risk factors (gender, bruxism, anxiety, and unilateral chewing) the difference was not significant ( *p*  = 0.3). 36 This reinforces the multifactorial character of this disorder. These studies, despite using reliable diagnostic criteria, investigated the relationship between tooth loss and TMD without considering their diagnostic subtypes. It is also worth noting that its cross-sectional research design precludes conclusions related to the association between factors. 

 The other four studies included in this review did not use validated diagnostic criteria and considered the diagnosis of TMD only by the presence of signs and symptoms, 32 33 34 35 in which the presence of joint sounds (clicking and crepitation), 32 33 34 35 pain in or around TMJ 32 33 34 and limited mouth opening 32 33 34 were the only signs and symptoms investigated. These articles also only evaluated unilateral or bilateral posterior tooth loss. 32 33 34 35 Of these, only one found a positive association between posterior tooth loss (Kennedy class I and II without considering the extent of loss) and the clinical presence of joint noise ( *p* <0.001) and TMJ pain ( *p* <0.01), 33 while in the others, 32 34 35 no statistical difference was found between tooth absence and the investigated signs and symptoms (joint noises, TMJ pain, and opening limitation). When comparing the two case–control studies included in the review, we observed divergent results, where Sarita et al did not find a significant difference in the presence of clicking and TMJ pain for the group with posterior tooth loss, but Fallahi et al finds this difference significantly. In the first study, 34 the presence of clicks was significant for the older group when compared to the younger ones ( *p* <0.001); these results suggest that the presence of clicks may be more related to age than to tooth loss, and it had already been observed in other studies. 43 44 These age-related adjustments were not made in the second study, 33 which may explain the divergence of results found along with other factors related to sample size, differences in sample characteristics, statistical analysis and others. 

A single longitudinal study was also included in this review. This study followed for 9 years a group with tooth loss and another with complete dentition and found no difference between the two groups in terms of prevalence, frequency, severity, or fluctuation of TMD signs/symptoms. In this study, the sample with tooth loss presented with the absence of posterior teeth with the presence of at least one premolar support bilaterally. The limitations of this study were related to the inclusion/exclusion criteria of the sample, lack of identification of confounding factors, loss of a considerable part of the sample, and it was classified as having a high risk of bias.

 In the inclusion criteria of the primary studies for this review, there were no restrictions related to the characteristics of tooth loss, i.e., articles that evaluated any type of tooth loss regardless of classification, quantity, location, or extent were considered. However, the classification of tooth loss used in these studies proved to be quite heterogeneous. Of the six included studies, only two of them used the Kennedy classification, 33 35 another one investigated the loss of one or more teeth without considering their quantity or location, 36 and the remaining three investigated the loss of posterior teeth with variations in their extension and location, taking into account the remaining posterior support units present. 30 32 34 35 37 It is possible that the use of a single classification standard would improve the comparison between them, however, in general, it was observed that the extent and location of tooth loss, despite being heterogeneous, do not seem to have influenced the final result, as most of them did not show a relationship between tooth loss and TMD. Most of these studies investigated loss of posterior teeth, perhaps this pattern of loss has been the most investigated, as there are records suggesting that the lack of teeth in the posterior region could generate overload and alterations in the TMJ, 45 46 although other studies have not found this relationship. 47 48 It is important to emphasize that there were no samples of completely edentulous patients in the studies included in this review. This probably occurred because they were eliminated at the beginning of the study because they did not meet the inclusion criteria of this review. 

 More recent reviews have encouraged clinicians to stop trying to find the role of occlusal characteristics in TMD etiology and to focus their efforts on integrating the critical study of scientific information already available with the clinic aspects. 21 25 The evidence on the association of tooth loss and TMD, although fragile, is the only one evidence the clinician has, and a lot of time and effort have already been wasted to clarify this relationship. The loss of teeth had been associated with TMD, but this association may be questionable when the evaluation is not controlled for other factors, as age, for example. 36 Recent and best designed studies show the influence of several others factors of association on beginning, worsening, and\or perpetuation of TMD. 20 49 50 It is known that tooth loss causes many damages to health and quality of life, so we have many reasons to rehabilitate patients, but the literature does not support us to associate the presence of signs and symptoms of TMD with this condition. Such association should be demystified among dentists, patients, and other health professionals. 

 Regarding methodological quality, only one study was evaluated with a moderate risk of bias and the others have been classified as having a high risk of bias. The confounding factors identifications was not reported in five studies. Then, the researchers did not perform strategies to deal with this confounding variable. Clinical features as age, psychosocial factors, and bruxism, were frequently encountered in TMD etiopathogenesis. 39 and these are variables that can influence the TMD diagnosis. 

 There are various ways to decrease the impact of confounding variables on the research, one of them is the statistical control. However, only Casa Nova et al performed it with a regression model. 36 Confounding factors represent a type of bias that needs to be measured and adjusted with adequate statistical analysis, 51 especially in TMD , due to the multiple factors involved in its etiopathogenesis. 

 The results of this review indicate that there is no quality evidence to confirm any association between the loss of one or more teeth and the diagnosis of TMD or the presence of signs and symptoms such as joint sounds, TMJ pain, and mouth opening restriction. In this sense, these findings corroborate with current literature in this field that shows the complexity of TMD as a disorder associated with multiple risk factors related to genetics, environment, psychosocial behavior, demographics, comorbidities 39 but without association with factors related to dental occlusion. 25

### Limitations

A substantial limitation in our review is related to the use of dental prostheses in the case samples of the included studies; most of the articles did not inform whether the sample group with tooth loss wore dental prosthesis or not in the period in which the study was carried out. Studies that brought this information were eliminated because we believe that the use of prosthesis would bring a bias to the study, however, these studies included in this review did not present this information clearly. In addition, inclusion and exclusion criteria and sample size calculation were not applied in most studies.

Another limitation found is that the primary studies showed high risk of bias (confounding bias and selection bias), absence of a standardized criteria to diagnose TMD, quantify and qualify tooth loss, which resulted in very heterogeneous data that made it impossible to carry out a meta-analysis. At the review level, there was no incomplete recovery of articles because all those selected or included were obtained and we believe that the reporting bias, if present, was minimal, not influencing the results of this study.

## Conclusion

There is no scientific evidence to support an association between one or more tooth loss and the presence of TMD signs and symptoms or diagnostic subgroups.
