# Supplementary material for: Updating The General Practitioner on The Association Between Teeth Loss and Temporomandibular Disorders: A Systematic Review
Source: Eur J Dent. 2022 Dec 27;17(2):296–309. doi: 10.1055/s-0042-1757209 (PMC10424267; doi:10.1055/s-0042-1757209)
Supplement: Supplementary file 1 — Supplementary Material [file 10-1055-s-0042-1757209-s2242065.pdf]

Supplementary Material Table S1 PRISMA 2020 item checklist

Section and Topic	Item #	Checklist item	Page
TITLE			
Title	1	Identify the report as a systematic review	1
ABSTRACT			
Abstract	2	See the PRISMA 2020 for Abstracts checklist	1
INTRODUCTION			
Rationale	3	Describe the rationale for the review in the context of existing knowledge.	1
Objectives	4	Provide an explicit statement of the objective (s) or question(s) the review addresses.	2
METHODS			
Eligibility criteria	5	Specify the inclusion and exclusion criteria for the review and how studies were grouped for the syntheses.	3
Information sources	6	Specify all databases, registers, websites, organizations, reference lists and other sources searched or consulted to identify studies. Specify the date when each source was last searched or consulted.	3
Search strategy	7	Present the full search strategies for all databases, registers and websites, including any filters and limits used.	S2 Table
Selection process	8	Specify the methods used to decide whether a study met the inclusion criteria of the review, including how many reviewers screened each record and each report retrieved, whether they worked independently, and if applicable, details of automation tools used in the process.	4
Data collection process	9	Specify the methods used to collect data from reports, including how many reviewers collected data from each report, whether they worked independently, any processes for obtaining or confirming data from study investigators, and if applicable, details of automation tools used in the process.	4
Data items	10a	List and define all outcomes for which data were sought. Specify whether all results that were compatible with each outcome domain in each study were sought (e.g. for all measures, time points, analyses), and if not, the methods used to decide which results to collect.	4
	10b	List and define all other variables for which data were sought (e.g. participant and intervention characteristics, funding sources). Describe any assumptions made about any missing or unclear information.	4
Study risk of bias assessment	11	Specify the methods used to assess risk of bias in the included studies, including details of the tool(s) used, how many reviewers assessed each study and whether they worked independently, and if applicable, details of automation tools used in the process.	4

(*Continued*)

 © 2022. The Author(s).

Supplementary Material Table S1 (*Continued*)

Section and Topic	Item #	Checklist item	Page
Effect measures	12	Specify for each outcome the effect measure (s) (e.g. risk ratio, mean difference) used in the synthesis or presentation of results.	NA
Synthesis methods	13a	Describe the processes used to decide which studies were eligible for each synthesis.	4
	13b	Describe any methods required to prepare the data for presentation or synthesis, such as handling of missing summary statistics, or data conversions.	4
	13c	Describe any methods used to tabulate or visually display results of individual studies and syntheses.	4
	13d	Describe any methods used to synthesize results and provide a rationale for the choice (s). If meta-analysis was performed, describe the model(s), method(s) to identify the presence and extent of statistical heterogeneity, and software package(s) used.	NA
	13e	Describe any methods used to explore possible causes of heterogeneity among study results.	NA
	13f	Describe any sensitivity analyses conducted to assess robustness of the synthesized results.	NA
Reporting bias assessment	14	Describe any methods used to assess risk of bias due to missing results in a synthesis (arising from reporting biases).	NA
Certainty assessment	15	Describe any methods used to assess certainty (or confidence) in the body of evidence for an outcome.	NA
RESULTS			
Study selection	16a	Describe the results of the search and selection process, from the number of records identified in the search to the number of studies included in the review, ideally using a flow diagram (see Figure 1).	Figure 1
	16b	Cite studies that met many but not all inclusion criteria ('near-misses') and explain why they were excluded.	S4 Table
Study characteristics	17	Cite each included study and present its characteristics.	5/6/Table 1
Risk of bias in studies	18	Present assessments of risk of bias for each included study.	Table 2, Table 3, Table 4
Results of individual studies	19	For all outcomes, present, for each study: (a) summary statistics for each group (where appropriate) and (b) an effect estimate and its precision (e.g. confidence/credible interval), ideally using structured tables or plots.	Table 1
Results of syntheses	20a	For each synthesis, briefly summarise the characteristics and risk of bias among contributing studies.	5
	20b	Present results of all statistical syntheses conducted. If meta-analysis was done, present for each the summary estimate and its precision (e.g. confidence/credible interval) and measures of statistical heterogeneity. If comparing groups, describe the direction of the effect.	NA

Supplementary Material Table S1 (*Continued*)

Section and Topic	Item #	Checklist item	Page
	20c	Present results of all investigations of possible causes of heterogeneity among study results.	NA
	20d	Present results of all sensitivity analyses conducted to assess the robustness of the synthesized results.	NA
Reporting biases	21	Present assessments of risk of bias due to missing results (arising from reporting biases) for each synthesis assessed.	5
Certainty of evidence	22	Present assessments of certainty (or confidence) in the body of evidence for each outcome assessed.	5
DISCUSSION			
Discussion	23a	Provide a general interpretation of the results in the context of other evidence.	7/8
	23b	Discuss any limitations of the evidence included in the review.	9
	23c	Discuss any limitations of the review processes used.	10
	23d	Discuss implications of the results for practice, policy, and future research.	11
OTHER INFORMATION			
Registration and protocol	24a	Provide registration information for the review, including register name and registration number, or state that the review was not registered.	1/3
	24b	Indicate where the review protocol can be accessed, or state that a protocol was not prepared.	3
	24c	Describe and explain any amendments to information provided at registration or in the protocol	3
Support	25	Describe sources of financial or non-financial support for the review, and the role of the funders or sponsors in the review.	12
Competing interests	26	Declare any competing interests of review authors.	12
Availability of data, code and other materials	27	Report which of the following are publicly available and where they can be found: template data collection forms; data extracted from included studies; data used for all analyses; analytic code; any other materials used in the review.	Supplementary Materials

 © 2022. The Author(s).

Supplementary Material Table S2 Search Strategy on each electronic database

Database	Search
EMBASE<br>August<br>2021	#1<br>“craniomandibular disorders”/exp OR “craniomandibular disorders” OR “temporomandibular joint disorders”/exp OR “temporomandibular joint disorders” OR “temporomandibular joint dysfunction syndrome”/exp OR “temporomandibular joint dysfunction syndrome” OR “disorders, temporomandibular joint” OR “joint disorder, temporomandibular” OR “joint disorders, temporomandibular” OR “myofascial pain dysfunction syndrome”/exp OR “myofascial pain dysfunction syndrome” OR “temporomandibular joint”/exp OR “temporomandibular joint” OR “tmj syndrome” OR “syndrome, tmj” OR “temporomandibular joint syndrome”/exp OR “temporomandibular joint syndrome” OR “joint syndrome, temporomandibular” OR “syndrome, temporomandibular joint” OR “craniomandibular disorder” OR “disorder, craniomandibular” OR “disorders, craniomandibular OR “craniomandibular diseases” OR “disease, craniomandibular” OR “diseases, craniomandibular”<br>#2<br>“tooth loss”/exp OR “tooth loss”/de OR “mouth, edentulous”/exp OR “mouth, edentulous“/de OR “jaw, edentulous”/exp OR “jaw, edentulous”/de OR “loss, tooth” OR “edentulous mouth” OR “edentulous mouths” OR “mouth, toothless” OR “toothless mouth” OR “edentulous jaw”/exp OR “edentulous jaw”/de OR “edentulous jaws” OR “jaws, edentulous” OR “edentulism”/exp OR “edentulism”/de OR “dental occlusion”/exp OR “dental occlusion”/de OR “edentulousness”/exp OR “edentulousness”/de
LILACS<br>August<br>2021	tw:((tw:(“Trastornos de la Articulación Temporomandibular” OR “Transtornos da Articulação Temporomandibular” OR “Síndrome de la Disfunción de Articulación Temporomandibular” OR “Síndrome da Disfunção da Articulação Temporomandibular” OR “Articulación Temporomandibular” OR “Articulação Temporomandibular”)) AND (tw:(“Pérdida de Diente” OR “Perda de Dente” OR “Boca Edéntula” OR “Boca Edêntula” OR “Arcada Edéntula” OR “Arcada Edêntula” “Arcada Desdentada” OR “Maxila Edentada” OR “Maxilar Desdentado” OR “Maxilar Edentado” OR “Maxilar Edêntulo” OR “Arcada Parcialmente Edêntula” OR “Arcada Parcialmente Edéntula” OR “Oclusión Dental” OR “Oclusão Dentária”))) AND (db:(“LILACS”))
LIVIVO<br>August<br>2021	“Tooth Loss” OR “Edentulism” OR “Edentulousness” AND ”Craniomandibular Disorders” OR “Temporomandibular Joint Disorders” OR “Temporomandibular Joint Dysfunction Syndrome” OR “Myofascial Pain Dysfunction Syndrome” OR “Temporomandibular Joint Syndrome” OR “Joint Syndrome, Temporomandibular” OR “Syndrome, Temporomandibular Joint” OR “Craniomandibular Disorder” OR “Craniomandibular Diseases”
PubMed<br>August 2021	(((((((((((((((((Tooth Loss[MeSH Terms]) OR (tooth loss[Title/Abstract])) OR (Mouth, Edentulous[MeSH Terms])) OR (Mouth, Edentulous[Title/Abstract])) OR (Jaw, Edentulous[MeSH Terms]))) OR (Loss, Tooth [Title/Abstract])) OR (Edentulous Mouth[Title/Abstract])) OR (Edentulous Mouths[Title/Abstract])) OR (Mouth, Toothless[Title/Abstract])) OR (Toothless Mouth[Title/Abstract])) OR (Edentulous Jaw[-Title/Abstract])) OR (Edentulous Jaws[Title/Abstract])) OR (Jaws, Edentulous[Title/Abstract])) OR (Edentulism[Title/Abstract])) OR (dental occlusion[MeSH Terms])) OR (dental occlusion[Title/Abstract])) AND ((((((((((((((((((((((((Craniomandibular Disorders[MeSH Terms]) OR (Craniomandibular Disorders [Title/Abstract])) OR (Temporomandibular Joint Disorders[MeSH Terms])) OR (Temporomandibular Joint Disorders[Title/Abstract])) OR (Temporomandibular Joint Dysfunction Syndrome[MeSH Terms])) OR (Temporomandibular Joint Dysfunction Syndrome[Title/Abstract])) OR (Disorders, Temporomandibular Joint[Title/Abstract])) OR (Joint Disorder, Temporomandibular[Title/Abstract])) OR (Joint Disorders, Temporomandibular[Title/Abstract])) OR (Myofascial Pain Dysfunction Syndrome,[Title/Abstract])) OR (Temporomandibular Joint[Title/Abstract])) OR (TMJ Syndrome[Title/Abstract])) OR (Syndrome, TMJ[-Title/Abstract])) OR (Temporomandibular Joint Syndrome[Title/Abstract])) OR (Joint Syndrome, Temporomandibular[Title/Abstract])) OR (Syndrome, Temporomandibular Joint[Title/Abstract])) OR (Craniomandibular Disorder[Title/Abstract])) OR (Disorder, Craniomandibular[Title/Abstract])) OR (Disorders, Craniomandibular[Title/Abstract])) OR (Craniomandibular Diseases[Title/Abstract])) OR (Disease, Craniomandibular[Title/Abstract])) OR (Diseases, Craniomandibular[Title/Abstract]))
Scopus<br>August<br>2021	TITLE-ABS-KEY (“Tooth Loss” OR “Mouth, Edentulous” OR “Jaw, Edentulous” OR “Loss, Tooth” OR “Edentulous Mouth” OR “Edentulous Mouths” OR “Mouth, Toothless” OR “Toothless Mouth” OR “Edentulous Jaw” OR “Edentulous Jaws” OR “Jaws, Edentulous” OR “Edentulism” OR “Dental occlusion” OR “Edentulousness”) AND TITLE-ABS-KEY (“Craniomandibular Disorders” OR “Temporomandibular Joint Disorders” OR “Temporomandibular Joint Dysfunction Syndrome” OR “Disorders, Temporomandibular Joint” OR “Joint Disorder, Temporomandibular” OR “Joint Disorders, Temporomandibular” OR “Myofascial Pain Dysfunction Syndrome” OR “Temporomandibular Joint” OR “TMJ Syndrome” OR “Syndrome, TMJ” OR “Temporomandibular Joint Syndrome” OR “Joint Syndrome, Temporomandibular” OR “Syndrome, Temporomandibular Joint” OR “Craniomandibular Disorder” OR “Disorder, Craniomandibular” OR “Disorders, Craniomandibular[“ OR “Craniomandibular Diseases” OR “Disease, Craniomandibular” OR “Diseases, Craniomandibular”) AND (LIMIT-TO (DOCTYPE, “ar”)) AND (EXCLUDE (SUBJAREA, “COMP”) OR EXCLUDE (SUBJAREA, “ENGI”) OR EXCLUDE (SUBJAREA, “HEAL”) OR EXCLUDE (SUBJAREA, “VETE”) OR EXCLUDE (SUBJAREA, “ARTS”))

 © 2022. The Author(s).

Supplementary Material Table S2 (*Continued*)

Database	Search
Web of Science August 2021	TÓPICO: (“Tooth Loss” OR “Mouth, Edentulous” OR “Jaw, Edentulous” OR “Loss, Tooth” OR “Edentulous Mouth” OR “Edentulous Mouths” OR “Mouth, Toothless” OR “Toothless Mouth” OR “Edentulous Jaw” OR “Edentulous Jaws” OR “Jaws, Edentulous” OR “Edentulism” OR “Dental occlusion” OR “Edentulousness”) *AND* TÓPICO: (”Craniomandibular Disorders” OR “Temporomandibular Joint Disorders” OR “Temporomandibular Joint Dysfunction Syndrome” OR “Disorders, Temporomandibular Joint” OR “Joint Disorder, Temporomandibular” OR “Joint Disorders, Temporomandibular” OR “Myofascial Pain Dysfunction Syndrome” OR “Temporomandibular Joint” OR “TMJ Syndrome” OR “Syndrome, TMJ” OR “Temporomandibular Joint Syndrome” OR “Joint Syndrome, Temporomandibular” OR “Syndrome, Temporomandibular Joint” OR “Craniomandibular Disorder” OR “Disorder, Craniomandibular” OR “Disorders, Craniomandibular [“ OR “Craniomandibular Diseases” OR “Disease, Craniomandibular” OR “Diseases, Craniomandibular”)
Google Scholar 2021	(“Temporomandibular Disorders” OR Temporomandibular Joint Disorders) AND (“tooth loss” OR “edentulous mouth”)
OpenGrey August 2021	“Tooth Loss” OR “Mouth, Edentulous” OR “Jaw, Edentulous” OR “Loss, Tooth” OR “Edentulous Mouth” OR “Edentulous Mouths” OR “Mouth, Toothless” OR “Toothless Mouth” OR “Edentulous Jaw” OR “Edentulous Jaws” OR “Jaws, Edentulous” OR “Edentulism” OR “Dental occlusion” OR “Edentulousness” AND ”Craniomandibular Disorders” OR “Temporomandibular Joint Disorders” OR “Temporomandibular Joint Dysfunction Syndrome” OR “Disorders, Temporomandibular Joint” OR “Joint Disorder, Temporomandibular” OR “Joint Disorders, Temporomandibular” OR “Myofascial Pain Dysfunction Syndrome” OR “Temporomandibular Joint” OR “TMJ Syndrome” OR “Syndrome, TMJ” OR “Temporomandibular Joint Syndrome” OR “Joint Syndrome, Temporomandibular” OR “Syndrome, Temporomandibular Joint” OR “Craniomandibular Disorder” OR “Disorder, Craniomandibular” OR “Disorders, Craniomandibular“ OR “Craniomandibular Diseases” OR “Disease, Craniomandibular” OR “Diseases, Craniomandibular”

 © 2022. The Author(s).

Supplementary Material Table S3 JBI critical appraisal checklist

JBI critical appraisal checklist for cohort studies	
1. Were the two groups similar and recruited from the same population?	Check the paper carefully for descriptions of participants to determine if patients within and across groups have similar characteristics in relation to exposure (e.g., risk factor under investigation). The two groups selected for comparison should be as similar as possible in all characteristics except for their exposure status, relevant to the study in question. The authors should provide clear inclusion and exclusion criteria that they developed prior to recruitment of the study participants.
2. Were the exposures measured similarly to assign people to both exposed and unexposed groups?	A high quality study at the level of cohort design should mention or describe how the exposures were measured. The exposure measures should be clearly defined and described in detail. This will enable reviewers to assess whether or not the participants received the exposure of interest.
3. Was the exposure measured in a valid and reliable way?	The study should clearly describe the method of measurement of exposure. Assessing validity requires that a 'gold standard' is available to which the measure can be compared. The validity of exposure measurement usually relates to whether a current measure is appropriate or whether a measure of past exposure is needed.<br>Reliability refers to the processes included in an epidemiological study to check repeatability of measurements of the exposures. These usually include intra-observer reliability and inter-observer reliability.
4. Were confounding factors identified?	Confounding has occurred where the estimated intervention exposure effect is biased by the presence of some difference between the comparison groups (apart from the exposure investigated/of interest). Typical confounders include baseline characteristics, prognostic factors, or concomitant exposures (e.g., smoking). A confounder is a difference between the comparison groups and it influences the direction of the study results. A high quality study at the level of cohort design will identify the potential confounders and measure them (where possible). This is difficult for studies where behavioral, attitudinal or lifestyle factors may impact on the results.
5. Were strategies to deal with confounding factors stated?	Strategies to deal with effects of confounding factors may be dealt within the study design or in data analysis. By matching or stratifying sampling of participants, effects of confounding factors can be adjusted for. When dealing with adjustment in data analysis, assess the statistics used in the study. Most will be some form of multivariate regression analysis to account for the confounding factors measured. Look out for a description of statistical methods as regression methods such as logistic regression are usually employed to deal with confounding factors/variables of interest.
6. Were the groups/participants free of outcome at the start of the study (or at the moment of exposure)?	The participants should be free of the outcomes of interest at the start of the study. Refer to the 'methods' section in the paper for this information, which is usually found in descriptions of participant/sample recruitment, definitions of variables, and/or inclusion/exclusion criteria.
7. Were the outcomes measured in a valid and reliable way?	Read the methods section of the paper. If for e.g., lung cancer is assessed based on existing definitions or diagnostic criteria, then the answer to this question is likely to be yes. If lung cancer is assessed using observer reported, or self-reported scales, the risk of over- or under-reporting is increased, and objectivity is compromised. Importantly, determine if the measurement tools used were validated instruments as this has a significant impact on outcome assessment validity.<br>Having established the objectivity of the outcome measurement (e.g., lung cancer) instrument, it's important to establish how the measurement was conducted. Were those involved in collecting data trained or educated in the use of the instrument/s? (e.g., radiographers). If there was more than one data collector, were they similar in terms of level of education, clinical or research experience, or level of responsibility in the piece of research being appraised?
8. Was the follow-up time reported and sufficient to be long enough for outcomes to occur?	The appropriate length of time for follow up will vary with the nature and characteristics of the population of interest and/or the intervention, disease or exposure. To estimate an appropriate duration of follow up, read across multiple papers and take note of the range for duration of follow up. The opinions of experts in clinical practice or clinical research may also assist in determining an appropriate duration of follow up. For example, a longer timeframe may be needed to examine the association between occupational exposure to asbestos and the risk of lung cancer. It is important, particularly in cohort studies that follow up is long enough to enable the outcomes. However, it should be remembered that the research question and outcomes being examined would probably dictate the follow up time.

 © 2022. The Author(s).

Supplementary Material Table S3 (*Continued*)

JBI critical appraisal checklist for cohort studies	
9. Was follow-up complete, and if not, were the reasons to loss to follow-up described and explored?	It is important in a cohort study that a greater percentage of people are followed up. As a general guideline, at least 80% of patients should be followed up. Generally a dropout rate of 5% or less is considered insignificant. A rate of 20% or greater is considered to significantly impact on the validity of the study. However, in observational studies conducted over a lengthy period of time a higher dropout rate is to be expected. A decision on whether to include or exclude a study because of a high dropout rate is a matter of judgement based on the reasons why people dropped out, and whether dropout rates were comparable in the exposed and unexposed groups.<br>Reporting of efforts to follow up participants that dropped out may be regarded as an indicator of a well conducted study. Look for clear and justifiable description of why people were left out, excluded, dropped out etc. If there is no clear description or a statement in this regards, this will be a 'No'.
10. Were strategies to address incomplete follow-up utilized?	Some people may withdraw due to change in employment or some may die; however, it is important that their outcomes are assessed. Selection bias may occur as a result of incomplete follow up. Therefore, participants with unequal follow up periods must be taken into account in the analysis, which should be adjusted to allow for differences in length of follow up periods. This is usually done by calculating rates which use person-years at risk, i.e. considering time in the denominator.
11. Was appropriate statistical analysis used?	As with any consideration of statistical analysis, consideration should be given to whether there was a more appropriate alternate statistical method that could have been used. The methods section of cohort studies should be detailed enough for reviewers to identify which analytical techniques were used (in particular, regression or stratification) and how specific confounders were measured.<br>For studies utilizing regression analysis, it is useful to identify if the study identified which variables were included and how they related to the outcome. If stratification was the analytical approach used, were the strata of analysis defined by the specified variables? Additionally, it is also important to assess the appropriateness of the analytical strategy in terms of the assumptions associated with the approach as differing methods of analysis are based on differing assumptions about the data and how it will respond.
Explanation of case–control studies critical appraisal	
1. Were the groups comparable other than presence of disease in cases or absence of disease in controls?	The control group should be representative of the source population that produced the cases. This is usually done by individual matching; wherein controls are selected for each case on the basis of similarity with respect to certain characteristics other than the exposure of interest. Frequency or group matching is an alternative method. Selection bias may result if the groups are not comparable.
2. Were cases and controls matched appropriately?	As in item 1, the study should include clear definitions of the source population. Sources from which cases and controls were recruited should be carefully looked at. For example, cancer registries may be used to recruit participants in a study examining risk factors for lung cancer, which typify population-based case–control studies. Study participants may be selected from the target population, the source population, or from a pool of eligible participants (such as in hospital-based case–control studies).
3. Were the same criteria used for identification of cases and controls?	It is useful to determine if patients were included in the study based on either a specified diagnosis or definition. This is more likely to decrease the risk of bias. Characteristics are another useful approach to matching groups, and studies that did not use specified diagnostic methods or definitions should provide evidence on matching by key characteristics. A case should be defined clearly. It is also important that controls must fulfil all the eligibility criteria defined for the cases except for those relating to the diagnosis of disease.
4. Was exposure measured in a standard, valid, and reliable way?	The study should clearly describe the method of measurement of exposure. Assessing validity requires that a "gold standard" is available to which the measure can be compared. The validity of exposure measurement usually relates to whether a current measure is appropriate or whether a measure of past exposure is needed.<br>Case–control studies may investigate many different "exposures" that may or may not be associated with the condition. In these cases, reviewers should use the main exposure of interest for their review to answer this question when using this tool at the study level.<br>Reliability refers to the processes included in an epidemiological study to check repeatability of measurements of the exposures. These usually include intraobserver reliability and interobserver reliability.
5. Was exposure measured in the same way for cases and controls?	As in item 4, the study should clearly describe the method of measurement of exposure. The exposure measures should be clearly defined and described in detail. Assessment of

(*Continued*)

 © 2022. The Author(s).

Supplementary Material Table S3 (*Continued*)

JBI critical appraisal checklist for cohort studies	
	exposure or risk factors should have been carried out according to same procedures or protocols for both cases and controls.
6. Were confounding factors identified?	Confounding has occurred where the estimated intervention exposure effect is biased by the presence of some difference between the comparison groups (apart from the exposure investigated/of interest). Typical confounders include baseline characteristics, prognostic factors, or concomitant exposures (e.g., smoking). A confounder is a difference between the comparison groups and it influences the direction of the study results. A high quality study at the level of case–control design will identify the potential confounders and measure them (where possible). This is difficult for studies where behavioral, attitudinal, or lifestyle factors may impact on the results.
7. Were strategies to deal with confounding factors stated?	Strategies to deal with effects of confounding factors may be dealt within the study design or in data analysis. By matching or stratifying sampling of participants, effects of confounding factors can be adjusted for. When dealing with adjustment in data analysis, assess the statistics used in the study. Most will be some form of multivariate regression analysis to account for the confounding factors measured. Look out for a description of statistical methods as regression methods such as logistic regression are usually employed to deal with confounding factors/ variables of interest.
8. Were outcomes assessed in a standard, valid and reliable way for cases and controls?	Read the methods section of the paper. If for e.g., lung cancer is assessed based on existing definitions or diagnostic criteria, then the answer to this question is likely to be yes. If lung cancer is assessed using observer reported, or self-reported scales, the risk of over- or under-reporting is increased, and objectivity is compromised. Importantly, determine if the measurement tools used were validated instruments as this has a significant impact on outcome assessment validity.<br>Having established the objectivity of the outcome measurement (e.g., lung cancer) instrument, it's important to establish how the measurement was conducted. Were those involved in collecting data trained or educated in the use of the instrument/s? (e.g., radiographers). If there was more than one data collector, were they similar in terms of level of education, clinical or research experience, or level of responsibility in the piece of research being appraised?
9. Was the exposure period of interest long enough to be meaningful?	It is particularly important in a case–control study that the exposure time is sufficient enough to show an association between the exposure and outcome. It may be that the exposure period may be too short or too long to influence the outcome.
10. Was appropriate statistical analysis used?	As with any consideration of statistical analysis, consideration should be given to whether there was a more appropriate alternate statistical method that could have been used. The methods section should be detailed enough for reviewers to identify which analytical techniques were used (in particular, regression or stratification) and how specific confounders were measured.<br>For studies utilizing regression analysis, it is useful to identify if the study identified which variables were included and how they related to the outcome. If stratification was the analytical approach used, were the strata of analysis defined by the specified variables? Additionally, it is also important to assess the appropriateness of the analytical strategy in terms of the assumptions associated with the approach as differing methods of analysis are based on differing assumptions about the data and how it will respond.
Explanation of analytical cross sectional studies critical appraisal	
1. Were the criteria for inclusion in the sample clearly defined?	The authors should provide clear inclusion and exclusion criteria that they developed prior to recruitment of the study participants. The inclusion/exclusion criteria should be specified (e.g., risk, stage of disease progression) with sufficient detail and all the necessary information critical to the study.
2. Were the study subjects and the setting described in detail?	The study sample should be described in sufficient detail so that other researchers can determine if it is comparable to the population of interest to them. The authors should provide a clear description of the population from which the study participants were selected or recruited, including demographics, location, and time period.
3. Was the exposure measured in a valid and reliable way?	The study should clearly describe the method of measurement of exposure. Assessing validity requires that a "gold standard" is available to which the measure can be compared. The validity of exposure measurement usually relates to whether a current measure is appropriate or whether a measure of past exposure is needed.<br>Reliability refers to the processes included in an epidemiological study to check repeatability of measurements of the exposures. These usually include intraobserver reliability and interobserver reliability.

 © 2022. The Author(s).

Supplementary Material Table S3 (*Continued*)

JBI critical appraisal checklist for cohort studies	
4. Were objective, standard criteria used for the measurement of condition?	It is useful to determine if patients were included in the study based on either a specified diagnosis or definition. This is more likely to decrease the risk of bias. Characteristics are another useful approach to matching groups, and studies that did not use specified diagnostic methods or definitions should provide evidence on matching by key characteristics.
5. Were confounding factors identified?	Confounding has occurred where the estimated intervention exposure effect is biased by the presence of some difference between the comparison groups (apart from the exposure investigated/of interest). Typical confounders include baseline characteristics, prognostic factors, or concomitant exposures (e.g., smoking). A confounder is a difference between the comparison groups and it influences the direction of the study results. A high quality study at the level of cohort design will identify the potential confounders and measure them (where possible). This is difficult for studies where behavioral, attitudinal, or lifestyle factors may impact on the results.
6. Were strategies to deal with confounding factors stated?	Strategies to deal with effects of confounding factors may be dealt within the study design or in data analysis. By matching or stratifying sampling of participants, effects of confounding factors can be adjusted for. When dealing with adjustment in data analysis, assess the statistics used in the study. Most will be some form of multivariate regression analysis to account for the confounding factors measured.
7. Were the outcomes measured in a valid and reliable way?	Read the methods section of the paper. If for e.g., lung cancer is assessed based on existing definitions or diagnostic criteria, then the answer to this question is likely to be yes. If lung cancer is assessed using observer reported, or self-reported scales, the risk of over- or under-reporting is increased, and objectivity is compromised. Importantly, determine if the measurement tools used were validated instruments as this has a significant impact on outcome assessment validity.<br>Having established the objectivity of the outcome measurement (e.g., lung cancer) instrument, it is important to establish how the measurement was conducted. Were those involved in collecting data trained or educated in the use of the instrument/s? (e.g., radiographers). If there was more than one data collector, were they similar in terms of level of education, clinical or research experience, or level of responsibility in the piece of research being appraised?
8. Was appropriate statistical analysis used?	As with any consideration of statistical analysis, consideration should be given to whether there was a more appropriate alternate statistical method that could have been used. The methods section should be detailed enough for reviewers to identify which analytical techniques were used (in particular, regression or stratification) and how specific confounders were measured.<br>For studies utilizing regression analysis, it is useful to identify if the study identified which variables were included and how they related to the outcome. If stratification was the analytical approach used, were the strata of analysis defined by the specified variables? Additionally, it is also important to assess the appropriateness of the analytical strategy in terms of the assumptions associated with the approach as differing methods of analysis are based on differing assumptions about the data and how it will respond.

 © 2022. The Author(s).

Supplementary Material Table S4 Excluded articles and reasons for exclusion

Author, Year	Reason for exclusion
Abdel-Fattah 1996[1]	1
Abdel-Raquim et al 1996[2]	1
Agerberg and Carlsson et al 1975[3]	1
Aldhalai et al 2017[4]	1
Almeida et al 2008[5]	2
Al-Shumailan et al 2015[6]	2
Amin et al 2019[7]	3
Bader 2020[8]	2
Banafa et al 2020[9]	2
Barghi et al 1987[10]	4
Bertram et al 2018[11]	4
Brito et al 2009[12]	5
Ciancaglini et al 1999[13]	1
Chairunnisa et al 2017[14]	2
Chatzopoulos et al 2017[15]	1
Costa et al 2012[16]	2
Czernaik et al 2018[17]	3
De Boever and Adriaens 1983[18]	4
Dervis 2004[19]	2
De Souza et al 2015[20]	2
Dulcic et al 2003[21]	2
Ekbäck et al 2013[22]	1
Elumalai et al 2018[23]	2
Garcia López et al 1997[24]	2
Garcia Rosalino (1) 2008[25]	3
Garcia Rosalino (2) 2008[26]	4
Gil and Nakamae 1998[27]	4
Gil and Nakamae 1999[28]	3
Gupta et al 2014[29]	2
Harriman et al 1990[30]	7
Hiltunen et al 1997[31]	3
Himawan et al 2007[32]	2
Holmlund and Axelsson 1994[33]	8
Ikebe et al 2008[34]	2
Javed et al 2020[35]	1
Katyayan et al 2016[36]	3
Kikiewicz 1978[37]	6
Kiverskari and Alanen 1985[38]	2
Kopp 1977[39]	2
Korzec and Jedrych 1982[40]	3
Malheiros et al 2016[41]	1
Manchikalapudi and Polasani 2017[42]	2
Michalak et al 2013[43]	4

 © 2022. The Author(s).

Supplementary Material Table S4 (*Continued*)

Author, Year	Reason for exclusion
Mundt et al 2005[44]	2
Nguyen et al 2017[45]	3
Pikhlak et al 2016[46]	6
Osterberg and Carlsson 1979[47]	2
Pullinger et al 1993[48]	2
Quaker 2011[49]	2
Reissman et al 2018[50]	2
Rodriguez et al 2013[51]	2
Sakurai et al 1988[52]	2
Sheety 2010[53]	2
Shet et al 2013[54]	2
Sipilla et al 2013[55]	2
Taboada Aranza et al 2004[56]	2
Tallents et al 2002[57]	2
Ternoven and Knuuttila 1988[58]	2
Uhac et al 2002[59]	2
Wang et al 2009[60]	2
Wilding and Owen 1987[61]	2
Witter et al 1988[62]	9
Witter et al 1994[63]	9

1, use of questionnaire and absence of clinical examination for the diagnosis of TMD ($n = 9$); 2, absence of a control group with complete dentition ($n = 33$); 3, prosthetic users in the edentulousness group ($n = 8$); 4, different evaluation outcome ($n = 6$); 5, children included in the sample ($n = 1$); 6, Congress abstract or literature review ($n = 2$); 7, edentulousness sample with associated comorbidities ($n = 1$); 8, sample submitted to previous treatments prior to the research ($n = 1$); 9, parcial duplication data of included study (2).

 © 2022. The Author(s).

Supplementary Material References of Excluded articles (listed in table S4)

1 Abdel-Fattah RA. Incidents of symptomatic temporomandibular (TM) joint disorders in female population with missing permanent first molar(s). Cranio 1996;14(01):55–62. Doi: 10.1080/08869634.1996.11745950

2 Abdel-Hakim AM, Alsalem A, Khan N. Stomatognathic dysfunctional symptoms in Saudi Arabian adolescents. J Oral Rehabil 1996;23 (10):655–661. Doi: 10.1046/j.1365-2842.1996.d01-181.x

3 Agerberg G, Carlsson GE. Symptoms of functional disturbances of the masticatory system. A comparison of frequencies in a population sample and in a group of patients. Acta Odontol Scand 1975;33(04):183–190. Doi: 10.3109/00016357509027560

4 Aldhalai MA, Alyami YA, Haider YM, et al. Prevalence and Severity of Temporomandibular Joint Disorders among Populations in Najran Province, Kingdom of Saudi Arabia. World J Dent 2017;8 (02):90–95

5 de Almeida LHM. Farias ABL. Soares MSM. de Almeida Cruz JS. da Cruz RES, de Lima M. Disfunção temporomandibular emidosos. Revista da Faculdade de Odontologia-UPF 2008;13(01):35–38

6 Al-Shumailan YR, et al. The Prevalence and Association of Signs and Symptoms of Temporomandibular Disorders with Missing Posterior Teeth in Adult Jordanian Subjects. Journal of the Royal Medical Services 2015;22:23–34

7 Amin M, Khan A, Khan MA. Frequency of common signs of temporomandibular disorders in patients With reduced occlusal support due to partial edentulism. Pak Oral Dent J 2019;39(02):206–211

8 Alzarea BK. Temporomandibular Disorders (TMD) in Edentulous Patients: A Review and Proposed Classification (Dr. Bader's Classification). J Clin Diagn Res 2015;9(04):ZE06–ZE09. Doi: 10.7860/JCDR/2015/13535.5826

9 Banafa A, Suominen AL, Sipilä K Factors associated with signs of temporomandibular pain: an 11-year-follow-up study on Finnish adults. Acta Odontol Scand 2020;78(01):57–63. Doi: 10.1080/00016357.2019.1650955

10 Barghi N, Aguilar T, Martinez C, Woodall WS, Maaskant BA. Prevalence of types of temporomandibular joint clickings in subjects with missing posterior teeth. J Prosthet Dent 1987;57 (05):617–620. Doi: 10.1016/0022-3913(87)90347-7

11 Bertram F, Hupp L, Schnabl D, Rudisch A, Emshoff R. Association Between Missing Posterior Teeth and Occurrence of Temporomandibular Joint Condylar Erosion: A Cone Beam Computed Tomography Study. Int J Prosthodont 2018;31(01):9–14. Doi: 10.11607/ijp.5111

12 Brito MI, Martínez TT, Rodríguez AMP. Saborit, TCí; Ramos, DA; MJ Rigau Factores de riesgo en pacientes con disfunción temporomandibular / Risk facts in patients with temporomandibular dysfunction. Rev Méd Electrón 2009;31(04):

13 Ciancaglini R, Gherlone EF, Radaelli G. Association between loss of occlusal support and symptoms of functional disturbances of the masticatory system. J Oral Rehabil 1999;26(03):248–253. Doi: 10.1046/j.1365-2842.1999.00368.x

14 Chairunnisa R, Sihombing R. The Association between Number of Tooth Loss, Tooth Loss Quadrants, and Occlusal Support with Temporomandibular Disorders in Partially Edentulous Patients. Advances in Health Science Research 2018;8:255–258

15 Chatzopoulos GS, Sanchez M, Cisneros A, Wolff LF. Prevalence of temporomandibular symptoms and parafunctional habits in a university dental clinic and association with gender, age, and missing teeth. Cranio 2019;37(03):159–167. Doi: 10.1080/08869634.2017.1399649

16 Costa MD, Junior F, Gontran da Rocha T, Santos CN. Evaluation of occlusal factors in patients with temporomandibular joint disorder. Dental Press J Orthod 2012;17(06):61–68

17 Czernaik CM, et al. Association between temporomandibular disorder symptoms and demographic, dental and behavioral factors in the elderly: a population-based cross-sectional study. Br J Pain São Paulo, 2018;1(03):223–30

18 De Boever JA, Adriaens PA. Occlusal relationship in patients with pain-dysfunction symptoms in the temporomandibular joints. J Oral Rehabil 1983;10(01):1–7. Doi: 10.1111/j.1365-2842.1983.tb00093.x

19 Dervis E. Changes in temporomandibular disorders after treatment with new complete dentures. J Oral Rehabil 2004;31(04): 320–326. Doi: 10.1046/j.1365-2842.2003.01245.x

20 de Sousa ST, de Mello VV, Magalhães BG, et al. The role of occlusal factors on the occurrence of temporomandibular disorders. Cranio 2015;33(03):211–216. Doi: 10.1179/2151090314Y.0000000015

21 Dulcić N, Pandurić J, Kraljević S, Badel T, Celić R Incidence of temporomandibular disorders at tooth loss in the supporting zones. Coll Antropol 2003;27(Suppl 2):61–67

22 Ekbäck G, Unell L, Johansson A, Ordell S, Carlsson GE. Changes in dental status and prevalence of symptoms related to temporomandibular disorders in 50- to 70-year-old subjects : longitudinal and cross-sectional results. Journal of craniomandibular function [Internet] 2013;5(04):317–31 Available from: *http://urn.kb.se/resolve?urn=urn:nbn:se:oru:diva-42582*

23 Elumalai M, Doraikannan SS, Indiran MA, Rathinavelu PK. Association of signs and symptoms of temporomandibular joint disorder between gender, partial edentulism, and morphological occlusion among dental patients in Chennai. Drug Invention Today. 2018;10:3617–3622

24 López EG, Fleites LMD, Alfonso JAD. Torre, A d la. Characterization of the sounds of the temporomandibular joint in partially edentelous patients. Rev cuba Ortod 1997;12(01):84–90

25 Garcia AR, Gallo AK, Zuim PR, Dos Santos DM, Antenucci RM. Evaluation of temporomandibular joint noise in partially edentulous patients. Acta Odontol Latinoam 2008;21(01):21–27

26 Garcia AR, Zuim PR, Goiato MC, et al. Effect of occlusion on joint sounds in asymptomatic individuals. Acta Odontol Latinoam 2008;21(02):135–140

27 GIL. C, NAKAMAE, AEM. Distúrbios craniomandibulares em pacientes edentados unilaterais inferiores com e sem próteses parciais removíveis (PPR): um estudo transversal utilizando o índice craniomandibular. Rev Odontol Univ Sao Paulo 1998;12 (02):189–197

28 Gil C, Nakamae AEM. Avaliação das disfunções craniomandibulares em pacientes parcialmente edentados unilaterais. [Internet] Rev Odontol Univ Sao Paulo 1999;13(03):275–282 Available from http://www.scielo.br/cgi-bin/fbpe/fbtext?got=last&pid=S0103-06631999000300012&usr=fbpe&lng=pt&seq=0103-0663-011&nrm=iso&sss=1&aut=71981947 citado 2022 set. 13

29 Gupta R, Malhi R, Patthi B, et al. Experience from Classroom Teaching to Clinical Practice Regarding Shortened Dental Arch (SDA) Concept Among Dentists - A Questionnaire Study. J Clin Diagn Res 2016;10(12):ZC27–ZC32. Doi: 10.7860/JCDR/2016/20855.9045

30 Harriman LP, Snowdon DA, Messer LB, et al. Temporomandibular joint dysfunction and selected health parameters in the elderly. Oral Surg Oral Med Oral Pathol 1990;70(04):406–413. Doi: 10.1016/0030-4220(90)90199-3

31 Hiltunen K, Vehkalahti M, Ainamo A. Occlusal imbalance and temporomandibular disorders in the elderly. Acta Odontol Scand 1997;55(03):137–141. Doi: 10.3109/00016359709115406

32 Himawan L, Kusdhany L, Ariani N. Temporomandibular disorders in elderly patients. Med J Indones 2007;16(04):237–239. Doi: 10.13181/mji.v16i4.288

33 Holmlund A, Axelsson S. Temporomandibular joint osteoarthrosis. Correlation of clinical and arthroscopic findings with degree of molar support. Acta Odontol Scand 1994;52(04):214–218. Doi: 10.3109/00016359409029049

 © 2022. The Author(s).

34 Ikebe K, Hazeyama T, Iwase K, et al. Association of symptomless TMJ sounds with occlusal force and masticatory performance in older adults. J Oral Rehabil 2008;35(05):317–323. Doi: 10.1111/j.1365-2842.2007.01841.x
35 Javed M, Asim M Fahimullah, Afreen Z, Afreen A, Khalil A. ASSOCIATION OF TOOTH LOSS WITH TEMPOROMANDIBULAR DISORDERS. KMUJ [Internet]. 2020Mar.31 [cited 2022Sep.13];12(1):29–3. Available from: *https://www.kmuj.kmu.edu.pk/article/view/196587*
36 Katyayan PA, Katyayan MK, Patel GC. Association of edentulousness and removable prosthesis rehabilitation with severity of signs and symptoms of temporomandibular disorders. Indian J Dent Res 2016;27(02):127–136. Doi: 10.4103/0970-9290.183129
37 Kikiewicz D. Effect of tooth loss on the function of the temporomandibular joint. Czas Stomatol 1978;31(08):759–62. Polish. PMID: 278726.
38 Kirveskari P, Alanen P. Association between tooth loss and TMJ dysfunction. J Oral Rehabil 1985;12(03):189–194. Doi: 10.1111/j.1365-2842.1985.tb00635.x
39 Kopp S. Clinical findings in temporomandibular joint osteoarthrosis. Scand J Dent Res 1977;85(06):434–443. Doi: 10.1111/j.1600-0722.1977.tb00577.x
40 Korzec J, Jedrych M. Relation between the type of tooth loss and the development of painless and painful forms of functional temporomandibular arthropathies. Protet Stomatol 1982;32(5–6):217–26. Polish. PMID: 6964797
41 Malheiros AS, Carvalhal ST, Pereira TL, et al. Association between Tooth Loss and Degree of Temporomandibular Disorders: A Comparative Study. J Contemp Dent Pract 2016;17(03):235–239. Doi: 10.5005/jp-journals-10024-1833
42 MANCHIKALAPUDI. G; POLASANI, LR. Correlation between posterior edentulousness and temporomandibular disorder in adult population: A case control study. IAIM 2017;4:143–150
43 Michalak M, Paulo M, Bożyk A, et al. Incidence of abnormalities in temporomandibular joints in a population of 1,100 urban and rural patients lacking teeth and other parafunctions in 2003-2008. An international problem. Ann Agric Environ Med 2013;20(01):86–90
44 Mundt T, Mack F, Schwahn C, et al. Gender differences in associations between occlusal support and signs of temporomandibular disorders: results of the population-based Study of Health in Pomerania (SHIP). Int J Prosthodont 2005;18(03):232–239
45 Nguyen MS, Jagomägi T, Nguyen T, Saag M, Voog-Oras Ü Occlusal Support and Temporomandibular Disorders Among Elderly Vietnamese. Int J Prosthodont 2017;30(05):465–470. Doi: 10.11607/ijp.5216
46 Pikhlak U, Ivanova E, Terentiev D. Evaluation of CMD frequency of occurrence in elderly patients permanently residing in a nursing home during routine dental examination. Int J Rheum Dis 2016;19:18
47 Osterberg T, Carlsson GE. Symptoms and signs of mandibular dysfunction in 70-year-old men and women in Gothenburg, Sweden. Community Dent Oral Epidemiol 1979;7(06):315–321. Doi: 10.1111/j.1600-0528.1979.tb01240.x
48 Pullinger AG, Seligman DA, Gornbein JA. A multiple logistic regression analysis of the risk and relative odds of temporomandibular disorders as a function of common occlusal features. J Dent Res 1993;72(06):968–979. Doi: 10.1177/00220345930720061301
49 Quaker AS. Consequences of Tooth Loss on Oral Function and Need for Rep Lacement of Missing Teeth Among Patients Attending Muhimbili Dental Clinic. Published online 2011 Available from: *https://core.ac.uk/download/pdf/11307459.pdf*
50 Reissmann DR, Anderson GC, Heydecke G, Schiffman EL. Effect of Shortened Dental Arch on Temporomandibular Joint Intra-articular Disorders. J Oral Facial Pain Headache 2018;32(03):329–337. Doi: 10.11607/ofph.1910
51 Rodríguez ET, Espinosa IA, Mendoza VF, Vivas PAP, Desiderio EQ, Jackeline NF Factores de riesgo asociados a trastornos temporomandibulares. Rev Cubana Estomatol [Internet]. 2013 Dic [citado 2022 Sep 13]; 50(4): 364–373. Disponible en: *http://scielo.sld.cu/scielo.php?script=sci_arttext&pid=S0034-75072013000400004&lng=es*
52 Sakurai K, Giacomo TS, Arbree NS, Yurkstas AA. A survey of temporomandibular joint dysfunction in completely edentulous patients. J Prosthet Dent 1988;59(01):81–85. Doi: 10.1016/0022-3913(88)90115-1
53 Shetty R. Prevalence of signs of temporomandibular joint dysfunction in asymptomatic edentulous subjects: a cross-sectional study. J Indian Prosthodont Soc 2010;10(02):96–101. Doi: 10.1007/s13191-010-0018-9
54 Shet RG, Rao S, Patel R, Suvvati P, Sadar LR, Yadav RD. Prevalence of temporomandibular joint dysfunction and its signs among the partially edentulous patients in a village of North Gujarat. J Contemp Dent Pract 2013;14(06):1151–1155. Doi: 10.5005/jp-journals-10024-1466
55 Sipilä K, Näpänkangas R, Könönen M, Alanen P, Suominen AL. The role of dental loss and denture status on clinical signs of temporomandibular disorders. J Oral Rehabil 2013;40(01):15–23. Doi: 10.1111/j.1365-2842.2012.02345.x
56 Taboada OA, Gutiérrez YLG, Aranza ST, Mendoza VMN. Prevalence of signs and symptoms of temporomandibular joint disorders in a group of older adults. Rev ADM 2004;61(04):125–129
57 Tallents RH, Macher DJ, Kyrkanides S, Katzberg RW, Moss ME. Prevalence of missing posterior teeth and intraarticular temporomandibular disorders. J Prosthet Dent 2002;87(01):45–50. Doi: 10.1067/mpr.2002.121487
58 Tervonen T, Knuuttila M. Prevalence of signs and symptoms of mandibular dysfunction among adults aged 25, 35, 50 and 65 years in Ostrobothnia, Finland. J Oral Rehabil 1988;15(05):455–463. Doi: 10.1111/j.1365-2842.1988.tb00181.x
59 Uhac I, Kovac Z, Vukovojac S, Zuvić-Butorac M, Grzić R, Delić Z The effect of occlusal relationships on the occurrence of sounds in the temporomandibular joint. Coll Antropol 2002;26(01):285–292
60 Wang MQ, Xue F, He JJ, Chen JH, Chen CS, Raustia A. Missing posterior teeth and risk of temporomandibular disorders. J Dent Res 2009;88(10):942–945. Doi: 10.1177/0022034509344387
61 Wilding RJ, Owen CP. The prevalence of temporomandibular joint dysfunction in edentulous non-denture wearing individuals. J Oral Rehabil 1987;14(02):175–182. Doi: 10.1111/j.1365-2842.1987.tb00706.x
62 Witter DJ, van Elteren P, Käyser AF. Signs and symptoms of mandibular dysfunction in shortened dental arches. J Oral Rehabil 1988;15(05):413–420. Doi: 10.1111/j.1365-2842.1988.tb00177.x
63 Witter DJ, De Haan AF, Käyser AF, Van Rossum GM. A 6-year follow-up study of oral function in shortened dental arches. Part II: Craniomandibular dysfunction and oral comfort. J Oral Rehabil 1994;21(04):353–366. Doi: 10.1111/j.1365-2842.1994.tb01150.x

 © 2022. The Author(s).
